# *Cryptosporidium parvum* Infection in SCID Mice Infected with Only One Oocyst: qPCR Assessment of Parasite Replication in Tissues and Development of Digestive Cancer

**DOI:** 10.1371/journal.pone.0051232

**Published:** 2012-12-13

**Authors:** Sadia Benamrouz, Karine Guyot, Sophie Gazzola, Anthony Mouray, Thierry Chassat, Baptiste Delaire, Magali Chabé, Pierre Gosset, Eric Viscogliosi, Eduardo Dei-Cas, Colette Creusy, Valerie Conseil, Gabriela Certad

**Affiliations:** 1 Laboratoire Environnement & Santé, Faculté Libre des Sciences et Technologies de Lille, Université Lille Nord de France, Lille, France; 2 Laboratoire de Biologie et Diversité des Pathogènes Eucaryotes Emergents, Centre d'Infection et d'Immunité de Lille, Institut Pasteur de Lille, INSERM U1019, CNRS UMR 8402, Université Lille Nord de France, Lille, France; 3 Plateforme d'Expérimentations et de Hautes Technologies Animales, Institut Pasteur de Lille, Lille, France; 4 Service d'Anatomie et de Cytologie Pathologiques, Groupe Hospitalier de l'Université Catholique de Lille, Université Lille Nord de France, Lille, France; 5 Faculté de Pharmacie, Université Lille Nord de France, Lille, France; 6 Laboratoire de Parasitologie-Mycologie, Centre de Biologie et Pathologie, Centre Hospitalier Régional et Universitaire de Lille & Faculté de Médicine de Lille, Université Lille Nord de France, Lille, France; Institut national de la santé et de la recherche médicale - Institut Cochin, France

## Abstract

Dexamethasone (Dex) treated Severe Combined Immunodeficiency (SCID) mice were previously described as developing digestive adenocarcinoma after massive infection with *Cryptosporidium parvum* as soon as 45 days post-infection (P.I.). We aimed to determine the minimum number of oocysts capable of inducing infection and thereby gastrointestinal tumors in this model. Mice were challenged with calibrated oocyst suspensions containing intended doses of: 1, 10, 100 or 10^5^ oocysts of *C. parvum* Iowa strain. All administered doses were infective for animals but increasing the oocyst challenge lead to an increase in mice infectivity (P = 0.01). Oocyst shedding was detected at 7 days P.I. after inoculation with more than 10 oocysts, and after 15 days in mice challenged with one oocyst. In groups challenged with lower inocula, parasite growth phase was significantly higher (P = 0.005) compared to mice inoculated with higher doses. After 45 days P.I. all groups of mice had a mean of oocyst shedding superior to 10,000 oocyst/g of feces. The most impressive observation of this study was the demonstration that *C. parvum*-induced digestive adenocarcinoma could be caused by infection with low doses of *Cryptosporidium*, even with only one oocyst: in mice inoculated with low doses, neoplastic lesions were detected as early as 45 days P.I. both in the stomach and ileo-caecal region, and these lesions could evolve in an invasive adenocarcinoma. These findings show a great amplification effect of parasites in mouse tissues after challenge with low doses as confirmed by quantitative PCR. The ability of *C. parvum* to infect mice with one oocyst and to develop digestive adenocarcinoma suggests that other mammalian species including humans could be also susceptible to this process, especially when they are severely immunocompromised.

## Introduction

*Cryptosporidium* species are worldwide spread apicomplexan parasitic protists that infect mostly the gastrointestinal tract of fish, amphibians, reptiles, birds and more than 150 species of mammals including human beings [1]. The infection results from the ingestion of *Cryptosporidium* oocysts through the consumption of fecally contaminated food or water or through direct person*-*to-person or animal-to-person contact [2]. The severity of the disease in patients vary from asymptomatic to lethal cryptosporidiosis, depending on infecting species, their site of infection, the age and immune status of the host. Infected immunocompetent individuals frequently develop acute gastroenteritis while immunocompromised individuals become chronically and sometimes fatally affected [2].

Currently, more than 20 *Cryptosporidium* species are regarded as valid [3], and two major species, *Cryptosporidium parvum* and *C. hominis*, are responsible for most human cases of cryptosporidiosis [4]. A high infectious power of *Cryptosporidium* isolates from human or animal origin was reported [5]. Thus, in healthy volunteers with no serologic evidence of past infection with *Cryptosporidium*, an oral dose of 30 *C. parvum* oocysts caused infection [5]. The same group reported that *Cryptosporidium hominis* ID50 was 10 oocysts [4]. In addition, it was also reported that a single oocyst of *C. meleagridis* can produce a patent infection in steroid-treated C57BL/6 mice [6].

In order to investigate the biological and pathological divergences between *Cryptosporidium* species or strains and to contribute to the understanding of the dynamics of the infection, Certad and collaborators developed a reproducible animal model of chronic cryptosporidiosis using Dex-treated adult SCID mice [7]. Animals were inoculated either with *C. parvum* which parasitises the intestinal tract, or with *C. muris* which has a tropism for the stomach of mice. Unexpectedly, they found using this model that an inoculum of 10^5^ oocysts of *C. parvum* but not *C. muris* was able to induce the development of invasive digestive adenocarcinoma [7]. However, which is the minimun number of oocysts capable of producing both infection and digestive neoplasia in this model? The question is important as far as this model can be used to explore the phenotypic properties of *Cryptosporidium* samples isolated from human stools or environment (mainly water and food), where oocyst amounts can often be very low. In order to better describe our animal model, we explored the potential ability of freshly isolated *Cryptosporidium* oocysts to induce both patent infection and gastrointestinal neoplastic changes when administered at very low dose.

## Materials and Methods

### *Cryptosporidium parvum* oocysts

*C. parvum* IOWA oocysts were purchased from Waterbo*rne™, Inc.* (New Orleans, Louisiana). The stock solution of oocysts was stored in shipping medium (phosphate-buffered saline or PBS with penicillin, streptomycin, gentamycin, amphotericin B and 0.01% Tween 20) at 4°C until use. Absence of bacteria and fungi was assured by testing the oocyst suspensions on Plate Count Agar (37°C, 1 week) and on Sabouraud plates (37°C, 1 week).

### Animal source

CB17-SCID 6–7 week-old female mice were obtained from a colony bred and regularly controlled for assessing infections (including *Helicobacter* spp.) at the Pasteur Institute of Lille (France). Animals were maintained under aseptic conditions in an isolator during the whole experimentation as previously described [7], [8], [9], [10]. Animal experiments were performed in the animal facility of the Pasteur Institute of Lille (research accreditation number, A59107). The experimental protocol was approved by the French regional ethical committee (approval number CEEA 112011). Evaluation of aspects of animal's condition was performed regularly to detect suffering. Clinical signs that could constitute an endpoint included, but were not limited to: rapid or progressive weight loss, debilitating diarrhea, rough hair coat, hunched posture, lethargy or any condition interfering with daily activities (e.g. eating or drinking, ambulation, or elimination).

### Experimental design

SCID mice were administered with 4 mg/L of dexamethasone sodium phosphate (Dex) (Merck, Lyon, France) via drinking water [7], [11]. Dexamethasone administration started two weeks prior to oral inoculation with *Cryptosporidium* oocysts (see below) and was maintained during the whole experimentation. Dex-added water was replaced three times a week.

Oocysts were inoculated to mice by oral-gastric gavage using 18–20 gauge feeding tubes. Each mouse was inoculated with 200 µl of PBS containing different amount of oocysts: 1, 10, 100 or 10^5^. For each dose 4 to 8 mice were inoculated (group 1 to group 4). Negative control mice were inoculated with PBS (n = 4) or with an inoculum of 10^5^ heat inactivated oocysts (90°C, 15 min) (n = 4). After gavage mice were housed in sterile capped cages. Infected mice were individualized to avoid physical contact and minimize the risk of infection by cross-contamination and negative control mice were grouped. Mice were followed-up to 100 days P.I. for evaluation of infectivity and neoplastic lesions development.

### Preparation of calibrated oocyst suspensions

The oocyst concentration of the *C. parvum* Iowa stock solution was confirmed by measuring in triplicate 10 µl-aliquots. Sampled fractions were placed on a multi-well slide, allowed to dry and fixed with methanol. A direct immunofluorescence assay (DFA) using a FITC conjugate anti-*Cryptosporidium* monoclonal antibody (Cellabs Pty. Ldt., Croissy-Beaubourg, France) was done. Wells were examined at a magnification of ×400 and the fluorescing oocysts were counted in 10 randomly selected microscopic fields. Before inoculation, oocyst viability of the stock solution was estimated by a trypsin-taurocholate excystation test [12]. Based on the excystation rate (50%), serial dilutions were performed to prepare all the doses. The doses of ≤100 oocysts were prepared in 6 aliquots of 200-µl: 5 aliquots were verified to assess potential divergences with the intended inoculum and the last aliquot was inoculated to mice. Verification of the amount of oocysts in each aliquot was done by filtering samples through a 0.4 µm 25 mm black polycarbonate filter. Then, a DFA was done on the filter, as previously described. The entire filter was then mounted onto a glass slide with Citifluor mounting medium (Biovalley). Oocysts present on the whole surface of the filter were counted (at a magnification of 400) by manual scan on an epifluorescence microscope (Axioplan 2, Zeiss). The mean of infective oocysts counted after verification of aliquots is represented in Table 1.

**Table 1:** Experimental cryptosporidiosis in SCID mice: influence of inoculum size on infectivity and histopathological findings.

Group of mice	Intended infective oocyst dose	Mean of infective oocysts after verification of prepared doses (SD)	Number of infected mice/total mice per groupe (%)	Histopathological findings: score of severity^a^	
				Stomach	Ileo-caecal area
1	1	3.6+1.8	2/7 (28.5)^d^	2 to 5	2 to 3
2	10	11.6+3.6	6/8 (75)	2 to 4	2 to 3
3	100	47.2+25	7/7 (100)^e^	3 to 5	3
4	100,000	ND	4/4 (100)	3to 5	3 to 4
5^b^	0	ND	0/3 (0)^f^	0	0
6^c^	0	ND	0/3 (0)^f^	0	0

Euthanasia was done 45 to 100 days P.I.

a 0, no lesion; 1, inflammation and/or regenerative changes; 2, low grade intraepithelial neoplasia (LGIEN); 3, high grade intraepithelial neoplasia (HGIEN), carcinoma in situ (limited to the epithelium) or intramucosal adenocarcinoma (invasion into the lamina propria through the basement membrane of glands). 4, submucosal adenocarcinoma when glands penetrate through the muscularis mucosa; 5, invasive adenocarcinoma with the invasion through the muscularis into the subserosa.

b Inoculation with PBS.

c Inoculation with 10^5^ heat-inactivated oocysts.

d One mouse found dead on day 2 P.I.

e One mouse found dead on day 5 P.I.

f One mouse found dead on day 1 P.I.

ND: Note done.

### Oocyst shedding assessment

To evaluate the oocyst shedding over the course of *Cryptosporidium* infection, freshly excreted fecal pellets were collected three times a week. Each mouse was transferred into an individual clean cage during 30–60 min. Feces were placed into a microtube and weighted before addition and homogenization in sterile MilliQ water. The detection and quantification of the oocyst shedding were done by immuno-magnetic separation (IMS) using Dynabeads anti-*Cryptosporidium* kit (Invitrogen, Cergy Pontoise, France) according to the supplier recommendation and as previously described [8], [10]. The oocyst suspension was lay down on immunofluorescence slides, and labeled with a FITC conjugate anti-*Cryptosporidium* monoclonal antibody (Cellabs Pty.Ldt., Croissy-Beaubourg, France). Enumeration of oocysts was performed on the whole surface of each well at a magnification of ×400 and the number of parasites was expressed per gram of feces. Infectivity was expressed as the proportion of animals that became infected at each dose.

### Histological analysis and immunohistochemistry

Periodically or when signs of imminent death appeared, mice were euthanatized by CO_2_ inhalation. Stomach and ileo-caecal regions were removed from each mouse, fixed in 10% neutral formalin and embedded in paraffin. Sections of 5 µm thick were stained by hematoxylin-eosin (Leica Autostainer-XL, Rueil-Malmaison, France) or used for immunohistochemistry.

Lesions at different sites were scored according to the “Nomenclature for Histologic Assessment of Intestinal Tumors in the Rodent”, and compared to the “Vienna classification” of the epithelial neoplasia of the gastrointestinal tract for humans”, as previously with slight modifications [8], [10]. Briefly: 0, no lesion; 1, inflammation and/or regenerative changes; 2, low grade intraepithelial neoplasia (LGIEN); 3, high grade intraepithelial neoplasia (HGIEN), carcinoma in situ (limited to the epithelium) or intramucosal adenocarcinoma (invasion into the lamina propria through the basement membrane of glands). 4, submucosal adenocarcinoma when glands penetrate through the muscularis mucosa; 5, invasive adenocarcinoma with the invasion through the muscularis into the subserosa.

The following histochemical and immunohistochemical analyses were performed using the BenchMark XT staining module (Ventana medical system, Meylan, France).

The Volgens-Gomori stain (Reticulin) [13] was employed for assessment of basement membrane integrity. A mouse monoclonal antibody to cytokeratin (undiluted) (AM071-5 M; Biogenex, Netherlands) was used to mark epithelial cells. Muscle fibers were stained using an anti-alpha smooth muscle actin monoclonal antibody (dilution 1∶100) (M0851; Dako, Denmark). Sections were examined using a Leica DMRB microscope equipped with a Leica digital camera connected to an Imaging Research MCID analysis system (MCID software, Cambridge, United Kingdom).

### Quantification of parasites in mouse tissue

#### DNA extraction from formalin-fixed paraffin-embedded tissue samples

Paraffin embeded tissues from ileo-caecal region from 17 mice were available for molecular analysis. DNA was extracted from a mixture of 2 sections of 25 µm of each tissue block. Histologic sections were processed by using xylene and ethanol for paraffin removal and were then rehydrated. To disrupt the oocysts, the samples were frozen (−80°C, 5 min) and thawed (99°C, 4 min) six times and were at last sonicated during 1 min. DNA was then extracted using the NucleoSpin tissue (Machery Nagel, Düren, Germany) following the manufacturer instructions except that the proteinase K digestion was performed overnight.

#### Real time quantitative PCR (qPCR) assays

Two TaqMan systems were developed: the *Cryptosporidium* Taqman assay and the in-house mouse Taqman assay. The primers and TaqMan probe used for the *Cryptosporidium* qPCR assay were those reported by Fontaine and Guillot (2002) [14] that positioned inside a specific 452 bp sequence (GenBank accession number AF188110) present in a single copy in the genome. The forward and reverse primers amplified a 138 bp fragment. The fluorescent TaqMan probe was labelled at the 5′ end with 6-carboxy-fluorescine (FAM) reporter dye and at the 3′ end with the black hole quencher 1 dye (BHQ-1). For the mouse Taqman assay, the target was the beta-actin gene (GenBank accession number AC144818), a single-copy-number housekeeping gene. The forward (5′-AGGCCAACCGTGAAAAGATG-3′) and reverse (5′-CTGAGAAGCTGGCCAAAGAGA-3′) primers were designed to amplify a 68-pb fragment. The fluorescent TaqMan probe (5′-CCCAGGTCAGTATCCCGGGTAACCC-3′) was labelled at the 5′ end with hexachloro-6-carboxy-fluorescein (HEX) reporter dye and at the 3′ end with the BHQ-1 quencher dye.

Each amplification was performed in a 25-µl reaction mixture that contained 1× iQ™ Supermix (Bio-Rad, France), 400 nM of each *Cryptosporidium* primer or 200 nM of each actin primer, 100 nM of the *Cryptosporidium* probe or 50 nM of the beta-actin probe and 5 µl of DNA sample. The qPCR reactions were performed on a Rotor-Gene 6000 instrument (Corbett Research, Qiagen, France) and included an initial denaturation at 95°C for 15 min followed by 49 cycles of denaturation at 95°C during 15 s and annealing/extension at 60°C during 1 min. Fluorescence acquisition was done immediately following each annealing/extension step.

All samples were measured in triplicate in each assay and negative controls without template were included in each PCR run. In order to circumvent the effect of PCR inhibitors, each DNA extract was tested pure or diluted 10 and 100 fold. Amplification and data analysis were performed with the Rotor-Gene 6000 Software.

#### Quantification standards and normalization of parasites in tissues

Specific external standards were constructed for both target genes of interest by cloning the fragment in a plasmid. The *Cryptosporidium* and tissue standard curves were then generated from six serial dilutions of plasmid DNA with known amounts of input copy numbers in each reaction. Linear regression of the standards dilution series and calculation of the corresponding R^2^ values were performed using the Rotor-gene software. Accuracy of absolute quantification relies on the assumption that DNA amplification efficiencies are similar between the standard and the tested samples. To test a possible influence of plasmid DNA in genomic DNA quantification, linearity and efficiency of both qPCR assays were also evaluated with both genomic *Cryptosporidium* and murine DNA. The number of *Cryptosporidium* genome and murine beta-actin gene copies in amplification reactions were automatically calculated by the software with reference to the external plasmidic standard curves. For accurate comparison of parasite infection in tissue samples, the amount of total host DNA in each sample was normalized by TaqMan qPCR of the murine beta-actin gene. Quantitative parasite burden data was therefore expressed as the ratio of the *Cryptosporidium* genome number over the mouse genome number for each sample. However, for easiest comparison between samples, variations in sample load were corrected by normalization of the *Cryptosporidium* genome copies to 10^6^ beta-actin copies.

### Statistical analysis

Fisher's exact test (two-tailed) was used to analyze infectivity (comparing groups infected with doses inferior to 10 or superior to 10 oocysts) or parasite loads in tissues. An analysis of variance (ANOVA) was conducted to account for the effects of relevant factors (inocula, day P.I.) and their interactions on daily oocyst excretion. Data analysis was performed with the statistical software Graphpad. Significance was defined as P<0.05.

## Results

In order to evaluate the infection susceptibility of mice challenged with calibrated suspensions containing different intended doses, the amount of oocyst in feces was estimated periodically. All administered doses containing different amounts of oocysts revealed to be infective for Dex-treated SCID mice but increasing the oocyst doses lead to an increase in the level of infectivity (P = 0.01). Two out of 7 mice (28.5%) inoculated with an intended dose of 1 oocyst, 6/8 mice (75%) receiving an intended dose of 10 oocysts, and all mice inoculated with intended doses of 10^2^ and 10^5^ viable oocysts developed chronic infection until euthanasia (45–100 days P.I.). None of the negative control mice discharged oocysts after oral inoculation with either PBS or 10^5^ heat-inactivated oocysts (Table 1).

**Figure 1:**
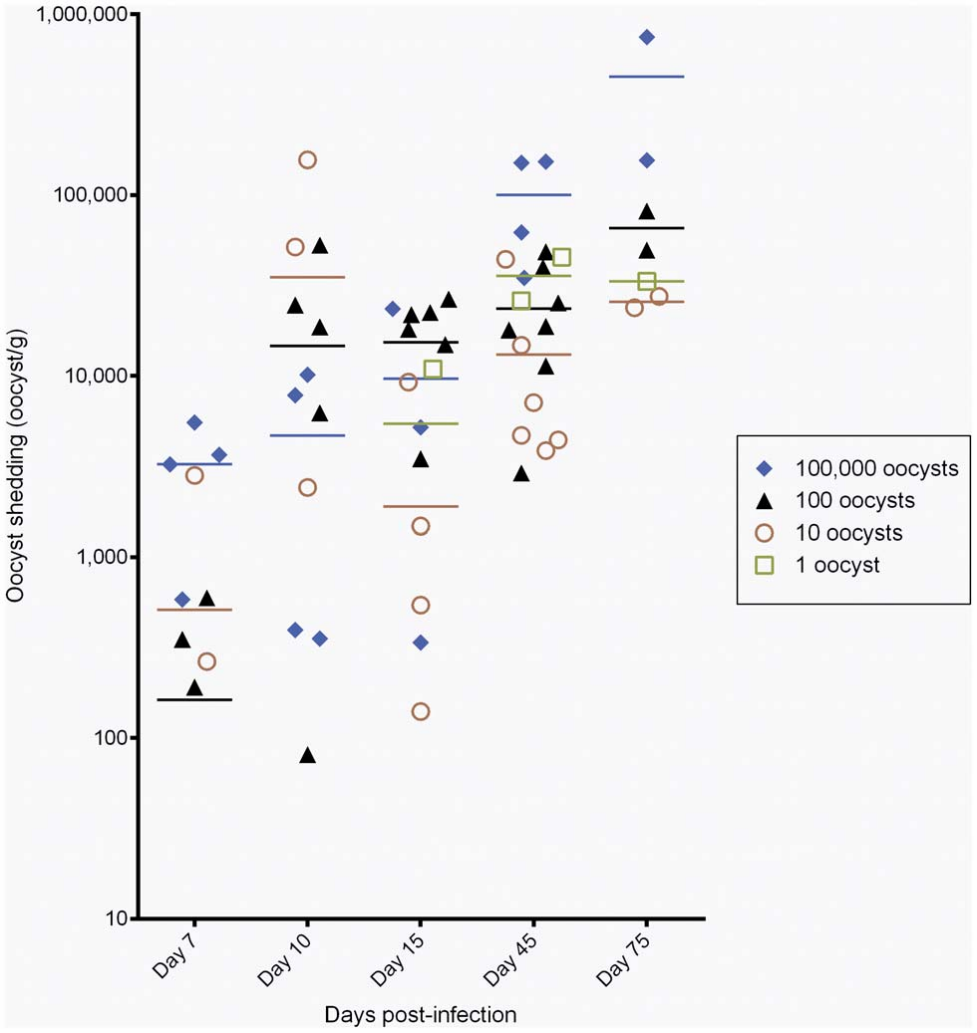
Pattern of oocyst shedding (oocyst/g feces) of Dex-treated SCID mice. Experimental groups were inoculated with intended doses of 1, 10, 100 and 10^5^ oocysts. Each point represents one mouse, the lines being the geometric means of oocyst shedding per group. Only animals with oocyst shedding at a precise moment of the day are represented. None of mice infected with one oocyst released parasites until day 15 (see material and methods for oocyst shedding assessment).

The pattern of oocyst shedding of the different groups is shown in Fig. 1. At day 7 P.I. oocyst shedding was detected in mice from groups 2, 3 and 4 (challenged with 10, 100 and 10^5^ oocysts) but not in animals from group 1 (challenged with one oocyst). Parasite shedding of animals from this latter group was detected for the first time after 15 days P.I. ANOVA analysis of the whole data set showed that the day P.I. and the inoculum size significantly influence the geometric means of oocyst sheding (P = 0.02 and 0.005, respectively): at 75 days P.I., mice inoculated with intended doses of 1, 10, 100 and 10^5^ oocysts had a multiplication of 3.3, 3.25, 2.26 and 1.45 log respectively compared to initial inoculum. After 45 days post-infection, all groups of mice have a mean of oocyst shedding superior to 10,000 oocyst/g confirming the high proliferation rate of parasite growth at lower doses.

**Figure 2:**
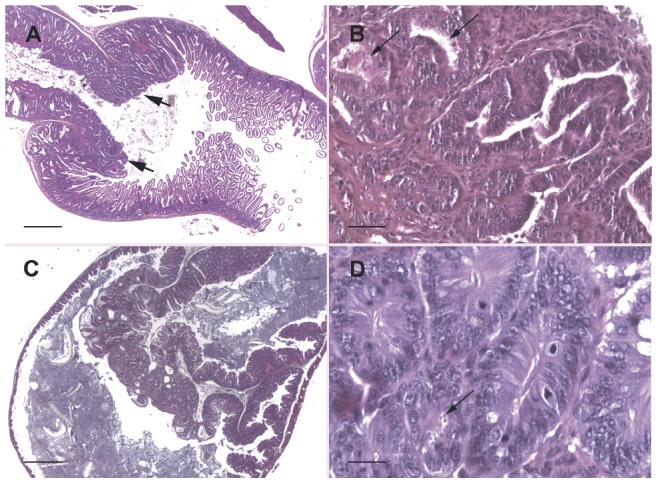
Gastro-intestinal neoplastic lesions in Dex-treated SCID mice infected with one oocyst of *C. parvum*. A. Invasive adenocarcinoma of the antropyloric region (arrows) in a mouse 100 days P.I.. Bar = 1250 µm (hematoxylin and eosin). B. Invasive adenocarcinoma of the antropyloric region in a mouse 100 days P.I. showing parasites inside the glands (arrows). Bar = 50 µm (hematoxylin and eosin). C. Adenoma of the ileo-caecal region in a mouse 45 days P.I.. Bar = 625 µm. D. High grade intraepithelial neoplasia in the ileo-caecal region in a mouse 45 days P.I. with numerous parasites (arrow) inside the glands. Bar = 25 µm (hematoxylin and eosin).

After histological examination of tissues, gastrointestinal neoplastic lesions (Table 1) were observed in all Dex-treated SCID mice infected by *C. parvum*, whatever the inoculum (Figure 2).

In the stomach, neoplastic lesions were localized in the antropyloric region and were detected as early as day 45 P.I. in all groups of infected mice. At day 45 P.I. these lesions were described as low grade intraepithelial neoplasia (LGIEN) or invasive adenocarcinoma for groups 1 and 2, and as high grade intraepithelial neoplasia (HGIEN) or invasive adenocarcinoma for groups 3 and 4 (Table 1). At day 100 P.I., we observed the presence of adenocarcinoma of the submucosa invading the external muscularis layer in the antro-pylorique region of a mouse inoculated with a single oocyst (Figures 2A, 2B).

In the ileo-caecal region, with intended doses of 1 and 10 oocysts a polypoid mucosa (adenoma) containing LGIEN and HGIEN lesions was observed respectively at 45 and 100 days P.I. (Figures 2C, 2D). With larger inoculum (100 oocysts) HGIEN was observed earlier (day 45 P.I.). Reticulin staining and cytokeratin immuno-labeling allowed the confirmation of the HGIEN: a fragmented basement membrane and neoplastic epithelial cells in the lamina propia were observed. These changes, which are typical of intramucosal adenocarcinoma, were observed in mice of group 3, after only 80 days P.I.. Neoplastic lesions seemed to be more severe in the stomach than in the ileo-caecal region.

For quantitative analysis of *Cryptosporidium* DNA in ileo-caecal tissues, 17 mice were selected. The standard curves generated for both *Cryptosporidium* and beta-actin showed a reproducible linear relationship between the Ct value and the log transformed number of copy over almost five orders of magnitude of DNA dilution. Correlation coefficient obtained by linear regression analysis of three independent experiments was R^2^ = 0.99 for both *Cryptosporidium* and mouse plasmids. DNA amplification efficiencies were 99% for *Cryptosporidium* and 89% for mouse. Standard curves were also performed with *Cryptosporidium* genomic DNA as well as mouse genomic DNA. For both, plotting of the delta Ct values (Ct plasmid DNA - Ct genomic DNA) against the logarithm of the dilution factors resulted in a curve slope lower than 0.1 (data not shown), demonstrating that plasmid DNA could be used to quantify genomic DNA.

The number of *Cryptosporidium* and the amount of murine DNA present in each sample were quantified by interpolation of the corresponding Ct values in the standard curves for *Cryptosporidium* DNA and for the murine beta-actin gene. Levels of parasite DNA in tissues of studied animals are shown in Table S1. In total, 14 out of 15 studied inoculated mice were colonized with *Cryptosporidium*.

The parasite load in tissues of mice inoculated with higher inoculum (10^5^) was higher when compared to mice inoculated with low doses (≤100 oocysts) (p<0.001). However, when comparing tissue loads at the same time P.I. (45 days) between the lowest and the maximal inoculum, mouse N°12, inoculated with 100,000 times more oocysts than mouse N°1, had only 3.6 fold more parasite loads. No *Cryptosporidium* DNA was found in one mouse (N°7) inoculated with 10 oocysts (Table S1). This mouse developed neither infection nor neoplastic lesions, as confirmed also by IMS-DFA.

For 6 samples, the *Cryptosporidium* qPCR was not positive for all 3 replicates assuming a Poisson distribution of template when detecting very low copy numbers of the target. For such samples, the obtained Ct values were between 39 and 40 signifying that the PCR reaction contained theoretically 1 genome copy. In fact, the detection limit of the assay was reached and we could not attempt quantification with an acceptable level of accuracy and reliability. Runs of *Cryptosporidium* qPCR were tested with samples at a lower dilution point in order to obtain lower Ct values. Unlikely, they were not validated due to a negative PCR result (Ct absence) or because the average shifts in Ct did not produce the expected change (respecting the 10-fold dilution).

Histological lesions were always associated with the presence of parasites as it was observed by microscopy (Figures 2B, 2D) and qPCR (Table S1). The DNA detection of parasites through qPCR corroborates that even mice with lowest parasites loads in tissues had neoplastic lesions. Neither parasites nor lesions were detected in negative control groups (mice inoculated with PBS without parasites or with heat-inactivated oocysts) (Tables 1, S1).

## Discussion

The present findings reveal that Dex-treated SCID mice are susceptible to infection with extremely low *C. parvum* inoculum sizes of 1 and 10 oocysts. These inoculated calibrated doses were assessed several times by microscopic observation (see Material and Methods and Table 1). This animal model of Dex treated SCID mice revealed to have a high sensitivity to *C. parvum* infection and could be very useful to assess the presence of this parasite in clinical or environmental samples in which oocysts rates can be very low (e.g. tap water, edible vegetables, insects).

We compared our results with data reported previously for another animal model of corticoid treated C57bl/6N mice infected with one oocyst of the same *C. parvum* Iowa isolate [15]. They found 17% of infectivity after inoculation with a single oocyst compared to 29% in our study [15]. As these authors also discussed, different reasons may explain the fact that not every mouse became infected with a single parasite [15]: the oocyst was not present in the inoculum due to the serial dilution method, the oocyst could remain in the gauge feeding tube during the inoculation, the oocyst was not able to reach the intestine or it was not viable at the time of inoculation (a viability rate of 50% was measured in the stock suspension).

Additionally, in our study animals challenged with low inoculum developed chronic infection and shed oocysts without stationary or decline phase until the end of the experiment.

The model of immunosupressed C57BL/6 adult mouse [15] was tested also for the propagation of *C. meleagridis* (species from birds) and 50% of the mice challenged with a single *C. meleagridis* oocyst became infected [16]. However, in despite of deep immunodepression of SCID mice, amplified further by Dex administering, these animals were apparently non susceptible to isolates of other *Cryptosporidium* species such as *C. meleagridis* (unpublished observations), *C. hominis* (from humans) or *C. molnari* (from the fish *Sparus auratus*) [10]. Thus, as it has been described for other opportunistic parasitic diseases, immunosuppression could be not enough to break the species barrier (or host-species specificity) [17].

Nevertheless, the most impressive observation of the present work is the demonstration that infection with low doses, even with only one oocyst of *C. parvum* can lead to the development of digestive invasive adenocarcinoma. We demonstrated before that our model of Dex-treated SCID mice was useful to assess the ability of *C. parvum* to induce digestive adenocarcinoma in animals inoculated with doses between 10^5^ and 10^8^ oocysts [7], [8], [10] but the minimum number of oocysts capable of inducing these kind of lesions was not known until now. In fact, as it was previously observed, after infection with higher inocula (10^5^–10^7^) of the *C. parvum* Iowa strain [7], [10], in the present study neoplastic lesions (LGIEN and HGIEN) were detected as early as day 45 P.I. both in the stomach and in the ileo-caecal region of Dex treated SCID mice challenged with intended doses of 100, 10 or even one oocyst, and these lesions could also evolve in an invasive adenocarcinoma progressing through all layers of the stomach and ileo-caecal region.

Consistently, we observed that in mice inoculated with low inocula the parasite excretion increased fast, reaching a mean of oocyst shedding of more than 10,000 oocyst/g of feces at 45 days P.I.. It seems that the few oocysts inoculated to mice had an important multiplication, and that an increase in oocyst inoculated doses raise the level of infectivity but not necessarily the shedding of parasites and the pathological outcome.

In experimental infections of immunocompetent animals, different observations have been reported. In cattle, after inoculation with as little as one red blood cell infected with *Babesia bovis*, another apicomplexan parasite, there was an increase in the prepatent period (as we also observed) but the high morbidity and mortality of animals was not altered in comparison to those infected with higher inocula [18]. These findings are in contrast to those for infections with the haemoparasite *Theileria parva*, where infection of cattle with a low inoculum resulted in decreased severity of disease and lower mortality [19]. Interestingly, in studies of *Eimeria* infection of chickens and rats, it was especially noticeable that with the greatest infecting dose, the number of oocysts produced per oocyst inoculated was smaller [20].

On the other hand, in our previous study, the oocyst shedding after inoculation with 10^5^ oocysts was much higher [8]. Nevertheless, natural lot to lot variability of the Iowa isolate was demonstrated before by a review of 22 dose response studies in a mouse model over a period of 3 years [21].

Our findings presented here confirm a great parasite amplification effect in mouse tissues after a low challenge of oocysts, and provide supplementary evidence of the role of *C. parvum* in the induction of digestive cancer. The DNA detection of parasites through qPCR corroborates that *C. parvum* is present in target organs and may lead to neoplasia. In some cases the amount of *Cryptosporidium* DNA present in tissues was not quantifiable in all three qPCR runs but it is well known that the isolation of genetic material from paraffin-embedded tissue sections can yield low amounts of DNA, which could be fragmented, degraded or folded with proteins [22]. It is also possible to have differences in the amounts of *Cryptosporidium* DNA extracted from one section of the organ to another one due to a variable distribution of parasites all along the gastro-intestinal tract. Additionally, inhibitions of the PCR reaction may occur due to the presence of large quantities of host DNA.

The ability of *C. parvum* to infect mice with one oocyst and to develop digestive adenocarcinoma suggests that other mammalian species including humans could be as susceptible to this process as Dex-treated SCID mice are, especially when they are severely immunocompromised.

In conclusion, the high infectious power of *Cryptosporidium* oocysts associated to its cancerogenic role was confirmed. For this reason, further studies should be done to explore this cancerogenic role of this parasite in human populations considering different factors: 1.The oocyst stage, a common contaminant in surface water world wide is resistant to disinfection with chlorine applied in drinking water treatment plants at standard concentrations [23]. 2. Different evidences show that low challenge of *Cryptosporidium* oocyst is infective to healthy humans. For instance, a mathematical model based on data from the Milwaukee outbreak suggested that some individuals developed cryptosporidiosis following the ingestion of only one oocyst [24]. Data from another study among healthy adults without evidence of past cryptosporidiosis showed that a low dose of *C. parvum* oocysts was sufficient to cause infection [5]. 3. A study reported that colon squamous cell carcinoma risk was significantly elevated among AIDS patients who had cryptosporidiosis [25]. Furthermore, a recent epidemiological study in Poland reported a frequency of 12.6% of cryptosporidiosis in patients with colorectal cancer [26].

## Supporting Information

Table S1 **Normalized quantification of parasites in ileo-caecal region of mice inoculated with different oocysts doses and euthanatized at different times post-infection.**
(DOC)
