# Supplementary material for: Cryptosporidium parvum Infection in SCID Mice Infected with Only One Oocyst: qPCR Assessment of Parasite Replication in Tissues and Development of Digestive Cancer
Source: PLoS One. 2012 Dec 13;7(12):e51232. doi: 10.1371/journal.pone.0051232 (PMC3521773; doi:10.1371/journal.pone.0051232)
Supplement: Table S1 — Normalized quantification of parasites in ileo-caecal region of mice inoculated with different oocysts doses and euthanatized at different times post-infection. (DOC) [file pone.0051232.s001.doc]

| Mouse no. | Inoculum | Day P.I. | Histopathological findings: score of severity^a^ | qPCR *Cryptosporidium* | | | qPCR mouse^b^ | | Normalized quantification of *Cryptosporidium* genome^c^ | |
| --- | --- | --- | --- | --- | --- | --- | --- | --- | --- | --- |
|  |  |  |  | Nb. of positive assay | Ct average | Ct standard deviation | Ct average | Ct standard deviation | average | standard deviation |
| 1 | 1 | 45 | 2 | 3 | 36.78 | 0.25 | 28.49 | 0.16 | 73.94 | 26.63 |
| 2 | 1 | 100 | 3 | 1 | 39.31 | - | 28.35 | 0.23 | nq | - |
| 3 | 10 | 45 | 2 | 2 | 39.29 | 0.34 | 26.96 | 0.28 | nq | - |
| 4 | 10 | 45 | 2 | 3 | 37.80 | 0.34 | 26.89 | 0.35 | 12.94 | 7.01 |
| 5 | 10 | 60 | 2 | 1 | 40.03 | - | 23.55 | 0.07 | nq | - |
| 6 | 10 | 60 | 2 | 1 | 39.48 | - | 26.53 | 0.19 | nq | - |
| 7 | 10 | 80 | 0 | 0 | - | - | 27.94 | 1.72 | 0 | - |
| 8 | 10 | 80 | 3 | 3 | 38.92 | 0.36 | 27.26 | 0.06 | 7.90 | 3.68 |
| 9 | 10 | 100 | 3 | 2 | 38.98 | 0.42 | 31.22 | 0.04 | nq | - |
| 10 | 100 | 45 | 3 | 2 | 39.68 | 0.80 | 29.71 | 0.10 | nq | - |
| 11 | 100 | 100 | 4 | 3 | 38.96 | 0.97 | 29.70 | 0.25 | 47.70 | 35.31 |
| 12 | 10^5^ | 45 | 3 | 3 | 34.73 | 0.24 | 28.25 | 0.15 | 267.19 | 70.49 |
| 13 | 10^5^ | 60 | 3 | 3 | 35.37 | 0.60 | 28.71 | 0.14 | 246.30 | 105.83 |
| 14 | 10^5^ | 80 | 4 | 3 | 34.43 | 0.17 | 26.88 | 0.07 | 153.28 | 38.52 |
| 15 | 10^5^ | 84 | 4 | 3 | 34.37 | 0.13 | 28.44 | 0.14 | 384.74 | 110.78 |
| 16 | PBS | 45 | 0 | 0 | - | - | 26.95 | 0.21 | 0 | - |
| 17 | 10^5^ heat inactivated | 100 | 0 | 0 | - | - | 29.22 | 0.27 | 0 | - |

^a^: 0, no lesion; 1, inflammation and/or regenerative changes; 2, low grade intraepithelial neoplasia (LGIEN); 3, high grade intraepithelial neoplasia (HGIEN), carcinoma in situ (limited to the epithelium) or intramucosal adenocarcinoma (invasion into the lamina propria through the basement membrane of glands). 4, submucosal adenocarcinoma when glands penetrate through the muscularis mucosa; 5, invasive adenocarcinoma with the invasion through the muscularis into the subserosa.

^b^ The 3 assays were positive for all mice.

^C^ Variations in sample load were corrected by normalization of the *Cryptosporidium* genome copies to 10^6^ beta-actin copies

nq: not quantifiable
